# NMP-7 inhibits chronic inflammatory and neuropathic pain via block of Cav3.2 T-type calcium channels and activation of CB2 receptors

**DOI:** 10.1186/1744-8069-10-77

**Published:** 2014-12-06

**Authors:** N Daniel Berger, Vinicius M Gadotti, Ravil R Petrov, Kevin Chapman, Philippe Diaz, Gerald W Zamponi

**Affiliations:** Department of Physiology and Pharmacology, Hotchkiss Brain Institute, Cumming School of Medicine, University of Calgary, Calgary, Canada; Core Laboratory for Neuromolecular Production, University of Montana, Missoula, MT USA

**Keywords:** T-type calcium channels, Neuropathic pain, Inflammatory pain, Cannabinoid receptors, Analgesia

## Abstract

**Background:**

T-type calcium channels and cannabinoid receptors are known to play important roles in chronic pain, making them attractive therapeutic targets. We recently reported on the design, synthesis and analgesic properties of a novel T-type channel inhibitor (NMP-7), which also shows mixed agonist activity on CB_1_ and CB_2_ receptors *in vitro*. Here, we analyzed the analgesic effect of systemically delivered NMP-7 (intraperitoneal (i.p.) or intragstric (i.g.) routes) on mechanical hypersensitivity in inflammatory pain induced by Complete Freund’s Adjuvant (CFA) and neuropathic pain induced by sciatic nerve injury.

**Results:**

NMP-7 delivered by either i.p. or i.g. routes produced dose-dependent inhibition of mechanical hyperalgesia in mouse models of inflammatory and neuropathic pain, without altering spontaneous locomotor activity in the open-field test at the highest active dose. Neither i.p. nor i.g. treatment reduced peripheral inflammation *per se*, as evaluated by examining paw edema and myeloperoxidase activity. The antinociception produced by NMP-7 in the CFA test was completely abolished in Ca_V_3.2-null mice, confirming Ca_V_3.2 as a key target. The analgesic action of intraperitoneally delivered NMP-7 was not affected by pretreatment of mice with the CB_1_ antagonist AM281, but was significantly attenuated by pretreatment with the CB_2_ antagonist AM630, suggesting that CB_2_ receptors, but not CB_1_ receptors are involved in the action of NMP-7 *in vivo*.

**Conclusions:**

Overall, our work shows that NMP-7 mediates a significant analgesic effect in a model of persistent inflammatory and chronic neuropathic pain by way of T-type channel modulation and CB_2_ receptor activation. Thus, this study provides a novel therapeutic avenue for managing chronic pain conditions *via* mixed CB ligands/T-type channel blockers.

## Background

Pathological chronic pain results from peripheral and central alterations in the nociceptive pathway. This persistent pain is difficult to treat and has a negative impact on a patient’s quality of life, as well as economic impacts associated with loss of productivity and cost of treatment. Chronic inflammatory and chronic neuropathic pain results from tissue injury and nerve injury, respectively, and both involve a peripheral and central sensitization event that culminate in pain from normally innocuous stimuli, allodynia, or exacerbated pain from otherwise mildly aversive stimuli, hyperalgesia [1]. The development of novel analgesics is paramount to the effective treatment of chronic pain, and the development of novel molecular entities targeting multiple mechanisms of pain neurobiology may be an attractive method to effectively mediate pain with lower compound doses, and reduced side effects [2].

Nociceptive transmission relies in part on low-voltage-activated T-type calcium channels that open in response to small membrane depolarizations [3]. T-type calcium channels are expressed along the primary afferent pain pathway [4–6] and these channels—in particular T-type channel subtype Ca_V_3.2—have shown potential as targets for analgesics [6]. Selective antisense oligonucleotide-mediated Ca_V_3.2 knockdown [7, 8] or inhibition of the T-type calcium channel by T-type channel modulators produce significant antinociceptive effects *in vivo* [9–13]. Painful diabetic neuropathies can be reversed by inhibiting T-type channels [14] as well as by Ca_V_3.2 antisense-mediated knockdown [15]. Ca_V_3.2 knockout mouse strains also show attenuated pain responses in the formalin-induced pain model [16]. Importantly, our laboratory has recently shown that interfering with Ca_V_3.2 channel trafficking mediates analgesia in mouse models of inflammatory and neuropathic pain [17].

**Figure 1:**
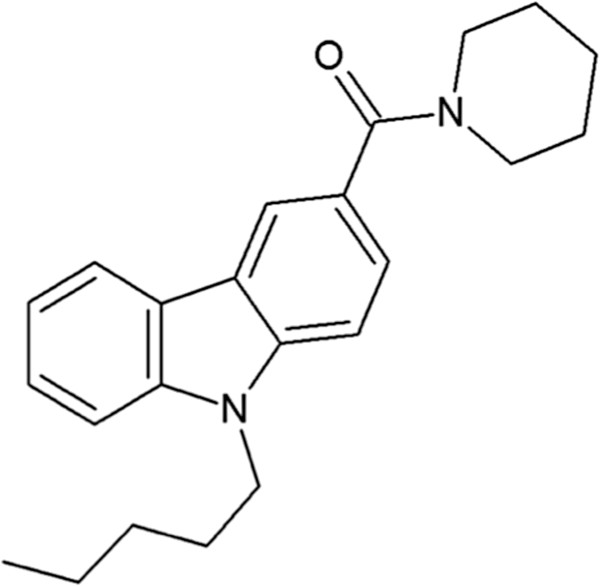
**Chemical structure of NMP-7.**

In addition to T-type calcium channels, the cannabinoid system has been recognized as a potential pharmacological target for chronic pain. The antinociceptive effects of the cannabinoid system make it an attractive target for relief of chronic pain, and randomized-controlled trials have indeed shown that cannabis use results in significant analgesia [18]. Interestingly, the endogenous cannabinoid anandamide also modulates T-type channels directly to produce thermal analgesia in a Ca_V_3.2-dependent manner [19, 20]. Additionally, both Δ^9^-THC and cannabidiol [21] or the endogenous cannabinoid anandamide and its derivatives [20–22] inhibit T-type channel activity. The use of such mixed CB/T-type calcium channel interacting compounds may provide a strategy for the development of better analgesics [23]. Indeed, combining different mechanisms of action in a single drug could possess several advantages that may include increased potency and effect duration, with a reduction of side effects and overall lower dose of the compound. In this context, we have previously reported that a series of mixed T-type channel inhibitors/cannabinoid receptor agonists, including NMP-7 (Figure 1), dose-dependently reduce formalin-induced nociception in mice [24]. NMP-7 was determined *in vivo* to have potent T-type channel blocking activity in electrophysiological measurements, and a 10-fold higher preference for CB_2_ receptors over CB_1_ receptors [24]. Here, we report on the antinociceptive action of NMP-7 in a model of persistent inflammatory pain and chronic neuropathic pain in mice, and identify *in vivo* the underlying mechanism of action of this compound.

## Results

### Analgesic effect of NMP-7 in persistent inflammatory pain and chronic neuropathic pain

**Figure 2:**
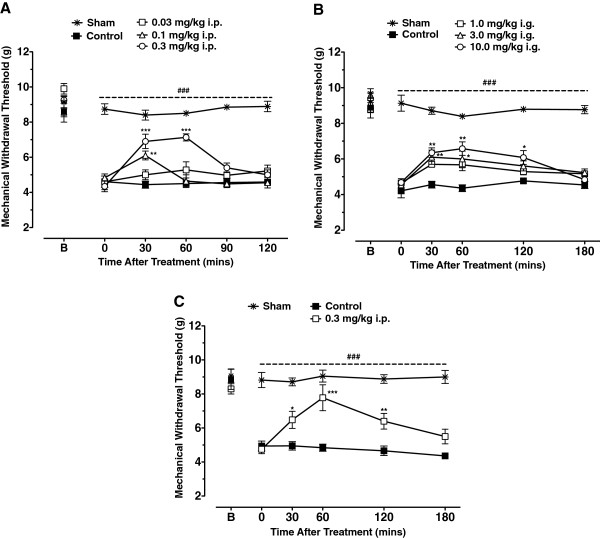
**Effect of intraperitoneal (A, C) or intragastric (B) treatment with NMP-7 on inflammatory pain and neuropathic pain.** Each point represents the mean ± SEM responses of 6–8 mice in Panels **A**, **B** and 6–12 mice in Panel **C**. Asterisks indicate significance relative to vehicle-treated (PBS +5% DMSO) control group. *p < 0.05, **p < 0.01 and ***p < 0.001 when compared to vehicle-treated controls, ^###^ *p* < 0.001 when comparing vehicle-treated control to sham group (Two-way ANOVA followed by a Tukey’s test).

To determine whether NMP-7 mediates an antinociceptive effect in mouse models of persistent inflammatory pain, we analyzed mechanical hypersensitivity in CFA-injected animals after systemic treatment with NMP-7. As shown in Figure 2A, B, mice injected with CFA developed mechanical hyperalgesia as indicated by a decrease in paw withdrawal thresholds when compared to the pre-CFA baseline levels of the vehicle control group (Two-way ANOVA, *p* < 0.0001). Three days after CFA injection, intraperitoneal treatment of mice with NMP-7 at 0.1 and 0.3 mg/kg significantly reversed mechanical hyperalgesia induced by CFA from 30 minutes up to 1 hour (*p* < 0.001 for 0.3 mg/kg) relative to vehicle-treated controls (Figure 2A). Similarly, intragastric NMP-7 treatment at 3 and 10 mg/kg significantly reversed mechanical hyperalgesia from 30 minutes up to two hours after treatment (*p <* 0.01 and *p* < 0.05 respectively for 10 mg/kg) (Figure 2B). In contrast, treatment of mice with vehicle control (PBS +5% DMSO) had no effect on the mechanical hyperalgesia induced by CFA injection.

**Figure 3:**
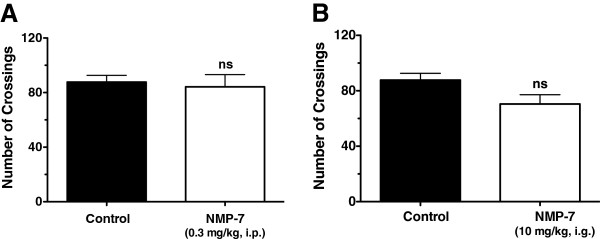
**Effect of intraperitoneal (A) and intragastric (B) NMP-7 treatment in the open field test.** Bars represent means ± SEM of total number of crossings of 10–14 animals. Control values (black bars) represent vehicle-treated group (PBS +5% DMSO) (Student’s *t*-test, ns = non-significant).

As seen in Figure 2C, partial sciatic nerve injury also produced significant mechanical hypersensitivity in mice when compared to sham-operated mice (*p <* 0.001). Intraperitoneal administration of NMP-7 (0.3 mg/kg) significantly decreased mechanical hyperalgesia from 30 minutes to two hours post-treatment, with a maximum effect observed at one hour post-treatment (Two-way ANOVA) (Figure 2C). Neither intraperitoneal (Figure 3A) nor intragastric (Figure 3B) treatment of mice with NMP-7 significantly altered the number of crossings in the open field test with the active doses. Taken together, these data show NMP-7 mediates a significant antinociceptive effect against CFA-induced persistent inflammatory pain and neuropathic pain with no nonspecific sedative or ataxic effects.

### NMP-7 does not affect inflammation per se

**Figure 4:**
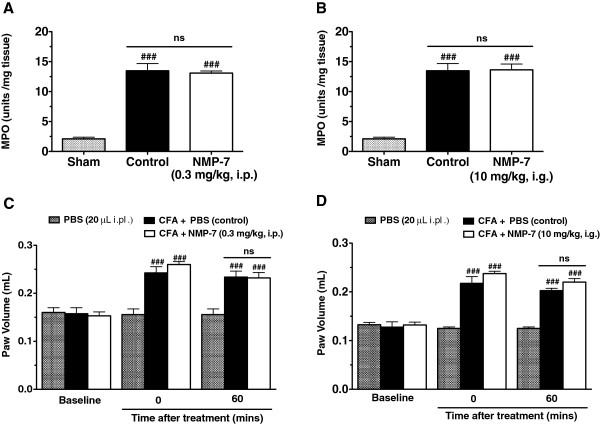
**Effect of intraperitoneal (A, C) or intragastric (B, D) treatment with NMP-7 on tissue myeloperoxidase (MPO) activity (Panel A and B) and paw volume (Panel C and D).** Bars represent mean ± SEM MPO units per mg of tissue (Panel **A**, **B**) and mean ± SEM of paw volumes (Panel **C**, **D**) of 7–10 mice when NMP-7 was delivered i.p. and 4–5 mice when delivered i.g. ^**###**^ *p <* 0.001 when compared to the non-inflamed group (20 μl of PBS injected intraplantarily), ns = non significant relative to the inflamed vehicle-treated controls (PBS +5% DMSO).

To rule out the possibility that NMP-7 may directly affect CFA-induced persistent inflammation we analyzed the effects of NMP-7 on paw volume and MPO activity. CFA injection 3 days prior to measurement produced significant paw edema (Two-way ANOVA, *p* < 0.0001) and neutrophil infiltration (One-way ANOVA, *p* < 0.0001) when compared to baseline measurements and PBS controls. Treatment of mice with NMP-7 at the active doses 0.3 mg/kg, i.p. (Figure 4A, C) or 10 mg/kg, i.g. (Figure 4B, D) yielded no significant decrease in peripheral myeloperoxidase levels (indicative of tissue neutrophil infiltration) 60 minutes post-treatment (Figure 4A, B), as well as no significant decrease in paw volume as assessed by plethysmometer (Figure 4C, D) relative to vehicle-treated control animals. These results show that NMP-7 does not mediate a direct peripheral anti-inflammatory effect.

### The mechanism of NMP-7 action involves Ca_V_3.2 T-type calcium channels

**Figure 5:**
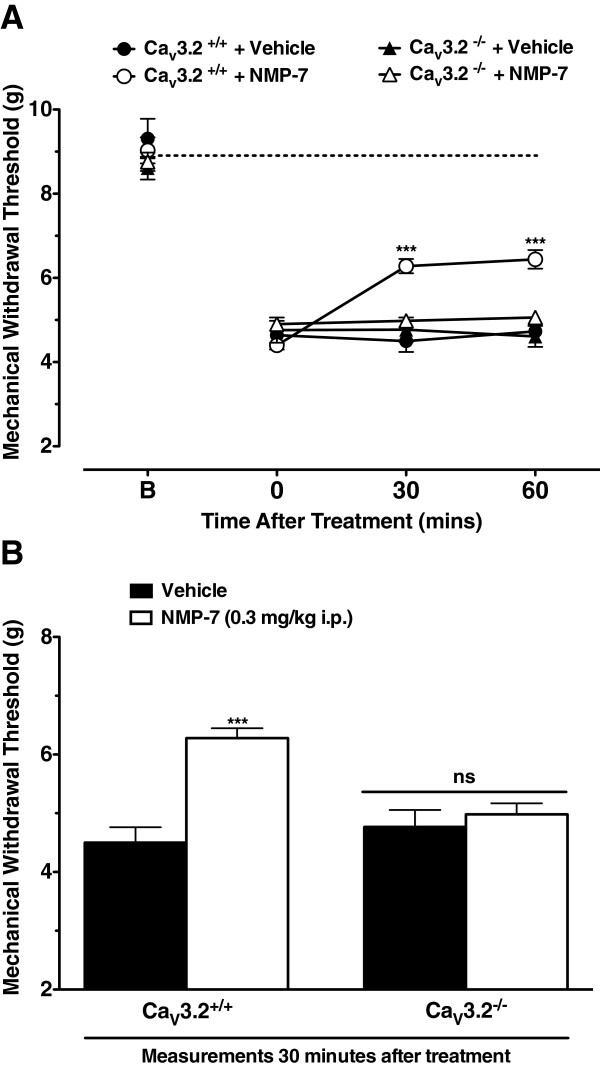
**Time-dependent effect (A) and bar representation (B) of NMP-7 delivered intraperitoneally to Ca**_**V**_ **3.2-null mice on inflammatory pain.** Each point (Panel **A**) represents the time dependent mean ± SEM of mechanical withdrawal thresholds of 7–14 wild type or Ca_V_3.2 null mice. Bars (Panel **B**) represent mean ± SEM responses of mechanical withdrawal threshold measurements taken 30 minutes after i.p. treatment of wild type and Ca_V_3.2 null mice with vehicle (PBS +5% DMSO, black bars) or NMP-7 (white bars) (n = 7-14). Asterisks indicate significance relative to vehicle control group, ***p < 0.001, ns = non significant (Three-way ANOVA for panel A and Two-way ANOVA for panel B, followed by a Tukey’s test).

To investigate whether the Ca_V_3.2 T-type calcium channel subtype is involved in NMP-7-mediated antinociception, the analgesic effect of NMP-7 delivered systemically (i.p.) was investigated in a Ca_V_3.2 knockout mouse strain. In response to CFA, these mice showed similar mechanical withdrawal thresholds relative to wild-type mice (Figure 5), as previously reported [17]. This is presumably due to compensation from other ion channels (for example sodium channels, although this has not been experimentally validated). Treatment with NMP-7 at 0.3 mg/kg, i.p. three days post-CFA injection produced a significant inhibition of mechanical hyperalgesia in wild type mice as expected (Three-way ANOVA, *p* < 0.0001), however Ca_V_3.2-null mice showed complete insensitivity to NMP-7 treatment (Figure 5A, B). These data validate Ca_V_3.2 as a primary target in the mechanism of NMP-7 action *in vivo*.

### The mechanism of NMP-7 action involves CB_2_, but not CB_1_ receptors

**Figure 6:**
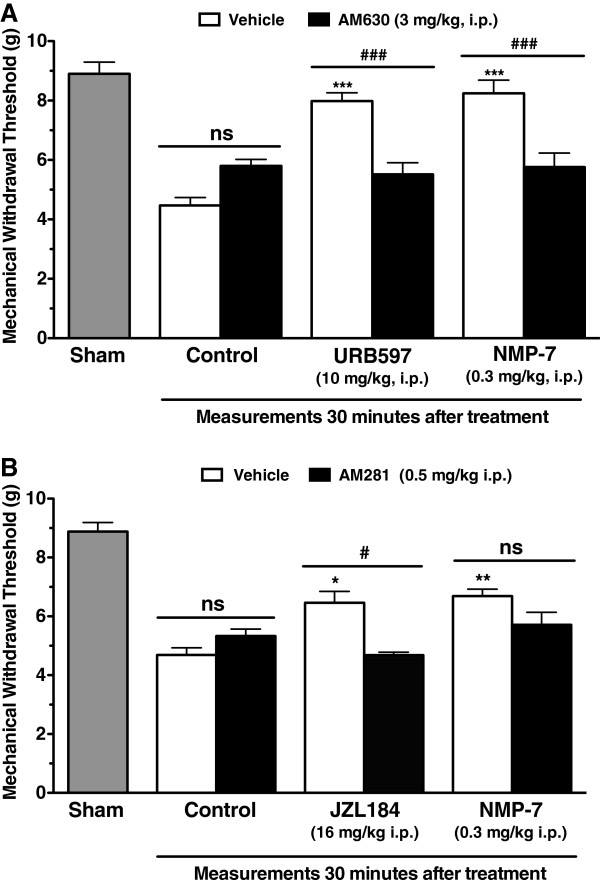
**Effect of pre-treatment of mice with selective CB**_**2**_ **(A) and CB**_**1**_ **(B) antagonists on the analgesic action of NMP-7.** Each bar represents mean ± SEM responses of 5–9 mice. Asterisks denote significance relative to vehicle-treated controls (PBS +5% DMSO), *p < 0.05, **p < 0.01 , ***p < 0.001. ^**#**^ *p* < 0.05, ^**###**^ *p* < 0.001 and ns = non significant when compared to antagonist-treated groups (Two-way ANOVA, followed by a Tukey’s test).

To determine the extent of CB receptor involvement in NMP-7’s mechanism of action *in vivo*, the selective CB_1_ antagonist AM281 and the CB_2_ antagonist AM630 were delivered to mice prior to NMP-7 treatment. Systemic pretreatment of mice with AM630 (3 mg/kg, i.p.) significantly attenuated the analgesic effects of NMP-7 (0.3 mg/kg, i.p.) at both 30 and 60 minutes post-treatment (Two-way ANOVA, *p* < 0.01) (Figure 6A). AM630 also reversed the analgesic effect of URB597 (10 mg/kg, i.p., an inhibitor of fatty acid amide hydrolase, the primary degradatory enzyme for the endocannabinoid anandamide), which was used as positive control (Two-way ANOVA, *p <* 0.01) (Figure 6A).

Systemic treatment of mice with the CB_1_ antagonist AM281 (0.5 mg/kg i.p.) did not reverse the antinociceptive action of NMP-7 (0.3 mg/kg, i.p.) at both 30 and 60 minutes post-treatment (Figure 6B). Yet, AM281 reversed the antinociceptive action of JZL184 (16 mg/kg, i.p., an irreversible inhibitor of monoacyglycerol lipase, the primary degradatory enzyme for endocannabinoid 2-arachidonoylglycerol) (Two-way ANOVA, *p* < 0.05) (Figure 6B), thus confirming that AM281 was indeed active *in vivo*. These data suggest that CB_2_, but not CB_1_ receptors are involved in the analgesic effect of NMP-7.

## Discussion

In this study, we have shown that the mixed CB agonist/T-type calcium channel inhibitor NMP-7 is efficacious in mediating analgesia in persistent inflammatory and chronic neuropathic pain through a mechanism that is dependent on Ca_V_3.2 calcium channels and CB_2_ receptors, but not CB_1_ receptors.

NMP-7 was previously characterized in an acute pain model in mice (i.e., injection of formalin into the hind paw) and was shown to attenuate both phases in a dose-dependent fashion when delivered intrathecally [24]. The effect in the second acute inflammatory phase of the formalin test suggested possible efficacy against persistent inflammatory pain. Indeed, as shown here, NMP-7 reversed the mechanical hyperalgesia in the CFA model of persistent inflammatory pain in a dose-dependent fashion for up to one hour when administered intraperitoneally, and two hours administered intragastrically. Furthermore, this compound reversed mechanical hyperalgesia following a peripheral nerve injury for up to two hours post-treatment. When administered systemically, NMP-7 resulted in significantly increased mechanical withdrawal thresholds without mediating non-specific sedative or ataxic effects at the active doses, as assessed by the open field test.

Systemic, peripheral and intrathecal administration of T-type calcium channel blockers such as mibefradil and ethosuximide have been previously shown to reverse mechanical and thermal hypersensitivity in response to nerve injury [11]. Inhibition of T-type channels or *in vivo* antisense-mediated knockdown produces antinociception in these and other models of chronic pain [7–16]. Conversely, increased T-type channel activity in the primary pain pathway occurs in models of chronic pain such as spinal nerve injury [25], peripheral nerve injury [26], colonic hypersensitivity [8] and diabetic neuropathy [27]. Altogether, these data indicate that T-type calcium channels mediate a pronociceptive role, and hence blockers that target these channels have the propensity to mediate analgesia. Along these lines, systemic and local administration of CB_2_ receptor agonists have been reported to produce analgesia in mice with peripheral nerve injuries, altogether indicating that both T-type calcium channels and CB_2_ receptors are important targets for treating neuropathic pain [28]. Here we show that a single compound, NMP-7, can target both of these pathways to trigger analgesic effects in not only neuropathic pain, but also hypersensitivity in response to CFA injection. Our data show that NMP-7 mediates its antinociceptive action largely through modulation of T-type calcium channels, and in part by CB_2_ receptor activation. Indeed, Ca_V_3.2-null mice were completely insensitive to the NMP-7 treatment and blocking CB_2_ receptors with AM630 also mediated a significant (albeit incomplete) reduction in the analgesic effects of this compound. This suggests that although NMP-7-mediated activation of CB_2_ receptors mediates analgesia, this may require the presence of functional Ca_V_3.2 channels. One possible explanation could be that CB_2_ receptors mediate their analgesic actions by inhibiting Ca_V_3.2 channels in afferent fibers. A number of second messenger pathways have indeed been shown to inhibit Ca_V_3.2 channel activity [3]. Hence, future studies should examine the possible coupling between CB_2_ receptors and T-type calcium channels in both expression systems and in dorsal root ganglion neurons. NMP-7 also mediated an antihyperalgesic effect for different lengths of time when administered intraperitoneally versus when administered intragastrically. This observed difference in the duration of NMP-7 effects is likely due to differential bioavailability of NMP-7; the pharmacokinetics of NMP-7 may differ between the two routes of administration.

CB_1_ receptors are expressed throughout the brain and the spinal cord, where they modulate neurotransmitter release such as inhibition of glutamate release by spinal cord interneurons [29, 30]. There is also evidence for CB_1_ expression on nociceptors in the periphery [31]. However, CB_1_ receptors almost exclusively mediate cannabis-related psychotropic effects, catalepsy and motor ataxia [29]. Hence, activation of CB_2_ receptors for pain relief is preferable to CB_1_ to avoid centrally-mediated psychotropic effects, and suppress the peripheral and central sensitization events that facilitate chronic pain development. NMP-7 fits this pharmacological profile as it binds to CB_2_ receptors with higher affinity than CB_1_ [24], and because of the inability of the CB_1_ receptor antagonist AM281 to prevent NMP-7 action *in vivo*.

## Conclusions

Taken together, NMP-7 mediates a pronounced analgesic effect that is dependent on T-type Ca_V_3.2 channels and CB_2_ receptors, and appears to cause no nonspecific motor effects at the therapeutic doses. Hence, T-type calcium channel blockers with CB_2_ agonist activity such as NMP-7 may be a viable avenue for the development of new chronic pain drugs.

## Methods

### Drugs and reagents

NMP-7 was synthesized at the Core Laboratory for Neuromolecular Production at the University of Montana. NMP-7 was dissolved in DMSO (to a maximum of 5%) and PBS. Selective CB_1_ antagonist AM281, irreversible inhibitor of monoacylglycerol lipase JZL184 [32, 33], selective CB_2_ antagonist AM630, and the irreversible inhibitor of fatty acid amide hydrolase URB597 [34] were provided by Cayman Chemical, and dissolved in phosphate buffered saline (PBS) and dimethyl sulfoxide (DMSO) to 5%. Complete Freund’s Adjuvant (CFA), o-Dianisidine and DMSO were supplied by Sigma Aldrich. The myeloperoxidase (MPO) assay standard was supplied by Calbiochem (EMD Millipore).

### Animals and drug treatment

Experiments were conducted in accordance with a protocol approved by the University of Calgary’s Institutional Animal Care and Use Committee, and all efforts were made to minimize animal suffering according to the policies and recommendations of the International Association for the Study of Pain. Adult male C57BL/6 J (wild-type) or *CACNA1H* knockout (Ca_v_3.2 null) mice (20-25 g, 6–8 weeks) were used and purchased from the Jackson Laboratory. Animals were housed at a maximum of five per cage (30 × 20 × 15 cm) with *ad libitum* access to food and water. Animals were kept in controlled temperature of 23 ± 1°C on a 12 h light/dark cycles (lights on at 7:00 a.m.). When drugs were delivered by intraperitoneal (i.p.) and intragastric (i.g.) routes, a constant volume of 10 ml/kg body weight was injected. Appropriate vehicle-treated groups were also assessed simultaneously. All compounds, including NMP-7, were dissolved in DMSO to a maximum 5% concentration, and PBS. Control animals received PBS +5% DMSO, and sham animals received no drug or vehicle. Choices of drug doses were based on previous literature [35] and from pilot experiments.

### CFA-induced persistent inflammatory pain

To induce inflammatory chronic pain and paw swelling, mice received a 20-μl injection of CFA subcutaneously in the plantar surface of the right hindpaw (intraplantarily, i.pl.). Control groups received 20 μl of PBS in the right hindpaw. This CFA treatment produces significant paw inflammation with accompanying hyperalgesia. Animals received NMP-7 either intraperitoneally (0.03 to 0.3 mg/kg) or intragastrically (1 to 10 mg/kg) 3 days post-CFA injection.

### Neuropathic pain induced by sciatic nerve injury

To induce chronic neuropathic pain, mouse sciatic nerves were ligated according to Malmberg and Basbaum [36]. Briefly, mice were anesthetized under 4% isoflurane, and held at 2.5% for the remainder of the surgery. The sciatic nerve was exposed, and the distal one-third to one-half section of the dorsal side of the nerve was transected and tightly tied with silk sutures. Sciatic nerves were exposed in sham-operated mice, but not tied. NMP-7 was administered intraperitoneally (0.3 mg/kg) two weeks post-surgery prior to testing mechanical withdrawal thresholds. Investigators were blind to treatment conditions when measurements were performed.

### Measurement of mechanical hyperalgesia

For both the inflammatory and neuropathic pain models, mechanical hyperalgesia was measured using the Dynamic Plantar Aesthesiometer (Ugo Basile, Varese, Italy). Animals were placed individually in a small, enclosed testing arena (20 cm × 18.5 cm × 13 cm, length × width × height) on top of a wire mesh grid. Mice were allowed to acclimate for a period of at least 90 minutes. The aesthesiometer device was positioned beneath the animal such that the filament was directly under the plantar surface of the ipsilateral hind paw. Each paw was tested three to four times per session, and measurements taken before the injuries were considered baseline measurements (B). Mice that had not developed persistent inflammatory or chronic neuropathic pain were excluded prior to treatment.

### Peripheral inflammation assays

In a different set of experiments from those described in the preceding section, peripheral inflammation was induced by a 20-μl injection of CFA subcutaneously in the plantar surface of the right hindpaw (i.pl.). Control groups received 20 μl of PBS in the right hindpaw. Mice received NMP-7 systemically (0.3 mg/kg, i.p. or 10 mg/kg, i.g.) 3 days post-CFA treatment, and paw volume was determined 60 minutes post-treatment by plethysmometer.

For the MPO assay, CFA- or PBS-treated back right hindpaws were collected 1 hour after NMP-7 administration at 0.3 mg/kg i.p. or 10 mg/kg i.g. Paws were homogenized with EDTA/NaCl buffer (pH 7.4), centrifuged at 4400 g (15 mins, 4°C), and the pellet was resuspended in ammonium bromide buffer (pH 5.4). The pellets were frozen and thawed three times in liquid nitrogen, and after the final thaw, re-centrifuged at 4400 g (15 mins, 4°C). 25 μl of the supernatant was assessed for MPO activity by absorbance at 650 nm, with o-Dianisidine and 0.3 mM H_2_O_2_ against an MPO standard.

### Open field test

Mouse ambulatory behavior was assessed in an open-field test as described previously [23]. The apparatus consisted of a wooden box measuring 40 × 60 × 50 cm with a frontal glass wall. The floor of the arena was divided into 12 equal squares and placed in a sound free room. Animals were placed in the rear left square and left to explore freely for 6 minutes, during which time the number of gridlines crossed with all paws (crossing) was counted. The apparatus was cleaned with a 70% alcohol solution and dried after each individual mouse session.

### Analysis of mechanism of action

To address the role played by T-type channels in the mechanisms by which NMP-7 produces antinociception, we tested NMP-7 delivered systemically at the active dose of 0.3 mg/kg in Ca_V_3.2 null mice in the CFA test. To investigate the involvement of CB_1_ receptor activation, the CB_1_ antagonist AM281 was administered alone and with NMP-7. JZL184 was used as a positive control, and was administered 15 minutes post-AM281 treatment in the same way as NMP-7. To investigate the extent of CB_2_ receptor involvement, the CB_2_ antagonist AM630 was delivered 15 minutes prior to NMP-7 treatment or URB597 treatment, which was used as a positive control.

### Data analysis

Each column or individual point (for line graphics) represents the mean ± SEM and is representative of at least 3 independent experimental runs. Data were evaluated by One-way, Two-way or Three-way analysis of variance (ANOVA) followed by the Tukey’s test, or alternatively a two-sample Student’s *t*-test. A value of *p* < 0.05 was considered to be significant.

## Abbreviations

CFA: Complete Freund’s Adjuvant
i.p.: Intraperitoneal
i.g: Intragastric
CB: Cannabinoid.
